# Phthalates in Fast Food: A Potential Dietary Source of Exposure

**DOI:** 10.1289/ehp.124-A191

**Published:** 2016-10-01

**Authors:** Wendee Nicole

**Affiliations:** Wendee Nicole was awarded the inaugural Mongabay Prize for Environmental Reporting in 2013. She writes for *Discover*, *Scientific American*, *National Wildlife*, and other magazines.

Many research studies have surveyed nutritional habits, but fewer have studied how food processing and packaging might introduce unwanted chemicals into foods. In this issue of *EHP*, researchers report that fast food consumption appears to be one source of exposure to the chemicals di(2-ethylhexyl) phthalate (DEHP) and diisononyl phthalate (DiNP).^1^

**Figure:**
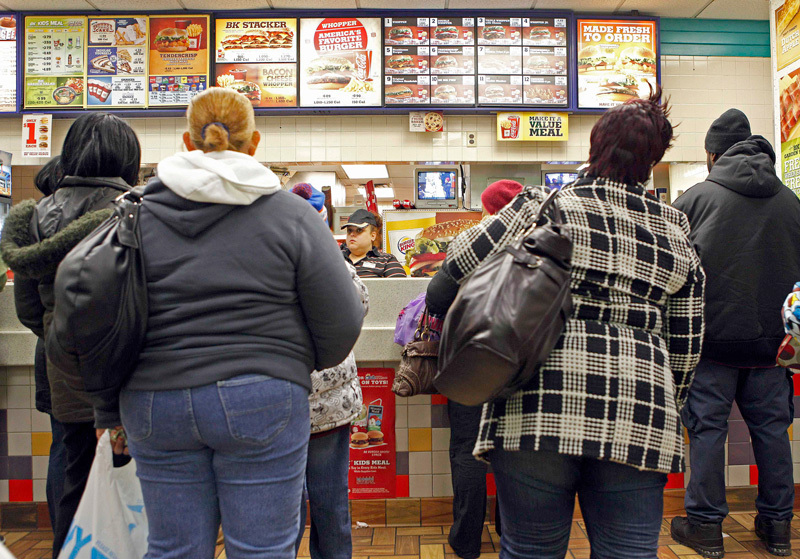
NHANES data indicate that non-Hispanic blacks are more likely than other racial/ethnic groups to eat fast food. This raises questions about disproportionate exposures to phthalates found in fast food. © Finbarr O’Reilly/Reuters

The authors used data from the National Health and Nutrition Examination Survey (NHANES) to estimate the percentage of individuals’ calories that came from fast food, fat intake attributable to fast food consumption, and fast food intake by food group. During NHANES interviews, respondents had reported their diet from the preceding 24 hours. Fast food was defined as food obtained from restaurants without waiter service and from pizza restaurants, as well as all carryout and delivery food.^2^ Regression analyses were used to determine associations between fast food consumption and urinary concentrations of DEHP metabolites, DiNP metabolites, and bisphenol A (BPA).

The final study population included nearly 9,000 people aged 6 years or older. Approximately one-third of people surveyed had eaten fast food in the preceding 24 hours. Study participants who ate fast food were more likely to be male, under age 40, and non-Hispanic black, and to have higher total calorie and total fat intake from fast food, compared with the general population.^1^

Fast food consumers had higher urinary levels of DEHP, DiNP, and BPA than non-consumers, although the differences in average urinary levels were small and for BPA were non-significant. When fast food intake was categorized by food group, DEHP metabolites were associated with intake of grains and “other” (a category that included vegetables, condiments, potato items, beverages, and more). DiNP metabolites were associated with intake of meat and grains.^1^

The authors also found that the associations between phthalates and fast food were not uniform across the population.^1^ They speculate that the pronounced association they saw between fast food consumption and DEHP in black consumers could reflect higher overall consumption of fast food and/or different food choices among this population. Prior research suggests that predominately black neighborhoods in urban areas have a greater density of fast food restaurants than white neighborhoods.^3^

“The fact that non-Hispanic blacks showed a steeper dose–response curve to fast food and DEHP is an important contribution to the environmental justice field since it suggests a potential connection between neighborhood environments, food choices, and phthalates exposure,” says lead author Ami Zota, an assistant professor of environmental and occupational health at George Washington University. Environmental justice research has found that minority populations often have greater environmental exposures to potentially harmful agents than other groups.^4^

The authors point to PVC tubing, vinyl gloves used for food handling, and food packaging as possible sources of phthalate contamination in fast food. DEHP is a ubiquitous high-molecular-weight phthalate that has been removed from some products due to concerns about potential adverse health effects.^5^ In some cases it is being replaced with DiNP.^2^

The study measured only exposures, not the potential health effects of fast food itself or of exposure to phthalates in fast food. At the same time, says Ruthann Rudel, director of research at the Silent Spring Institute, “One important implication of this finding is that dietary habits such as fast food intake may confound associations between urinary phthalate metabolite levels and health effects in epidemiology studies.” Rudel was not involved in the study.

“Dr. Zota has leveraged available resources from a nationally representative study population in a very savvy way to answer an interesting research question about chemical exposure via food packaging and processing,” says Megan Romano, a postdoctoral research associate in epidemiology at Brown University, who was not involved with the research. “The next steps are to better describe which types of foods have high levels of phthalate contamination and to identify the source of the phthalate contamination, be it processing or contact with food packaging.”
